# Psychological Drivers and Behavioral Outcomes of Fast Fashion Consumption: A Meta-Analytic

**DOI:** 10.12688/f1000research.170388.2

**Published:** 2026-02-10

**Authors:** Shu-Chuan Hsu, Ying-Kai Liao, Kuo-Chung Huang, Lun-Chuan Lin, Vo Thi Thinh, Wann-Yih Wu, Khemraj Sharma

**Affiliations:** 1 Department of Business Administration, Nanhua University, Dalin, Taiwan Province, 62249, Taiwan; 2 Department of International Business Administration, Nanhua University, Dalin, Taiwan Province, 62249, Taiwan; 3 International relations, Kalinga Institute of Industrial Technology, Bhubaneswar, Odisha, 751024, India

**Keywords:** Perceived scarcity, perceived quality, self-congruity, brand attitude, subjective norm, perceived behavioral control, consumption intention, brand loyalty, word-of-mouth, meta-analysis

## Abstract

This study employs a meta-analytic approach to synthesize empirical evidence on the psychological and behavioral determinants of fast fashion consumption. Integrating the Theory of Planned Behavior (TPB) with brand-related constructs—perceived scarcity, perceived quality, and self-congruity—this research examines how these factors shape consumer attitudes, subjective norms, perceived behavioral control, consumption intentions, brand loyalty, and word-of-mouth.

Using studies published between 2004 and 2024, a random-effects meta-analysis reveals that brand attitude is the strongest predictor of purchase intention, while self-congruity with fashion brands significantly enhances all TPB components. Perceived quality exerts a cross-cutting influence on both cognitive and social evaluations, reinforcing the multidimensional nature of consumer judgments.

The findings extend the TPB framework by embedding symbolic and perceptual brand dimensions, offering a more comprehensive explanatory model of fashion consumption. From a managerial perspective, the results suggest that marketing strategies emphasizing authentic scarcity cues and alignment with consumers’ self-identity can strengthen emotional attachment, perceived control, and loyalty. The study concludes with theoretical and practical implications for designing culturally sensitive and identity-driven branding strategies in the fast fashion sector.

## 1. Introduction

The rapid proliferation of fast fashion has significantly reshaped the landscape of global consumer culture. Driven by the principles of affordability, trend responsiveness, and accelerated product turnover, fast fashion brands have disrupted traditional apparel markets and deeply influenced consumption patterns, particularly among younger demographics. This evolving industry model satisfies consumers’ pursuit of immediacy and novelty, yet it simultaneously raises alarming concerns related to environmental degradation, ethical production, and the overextension of natural resources. Moreover, the psychological mechanisms underlying fast fashion consumption—characterized by emotional impulses, brand identification, and peer influence—are far more complex than traditional rational decision-making models can fully explain. Although the TPB has been widely employed to interpret behavioral intentions in consumption studies, its core framework, which focuses on attitude, subjective norms (SN), and perceived behavioral control (PBC), may insufficiently capture the symbolic and affect-driven aspects of consumer-brand interaction in fast fashion contexts. In particular, variables such as perceived scarcity (PS), perceived quality (PQ), and self-congruity with fast fashion brands—elements often shaped through marketing practices like limited-edition releases, celebrity collaborations, and influencer endorsement—remain underexplored and inconsistently measured across empirical studies.

The motivation for this study arises from several critical research gaps and practical needs. First, past investigations into fast fashion consumption have been largely siloed by region or culture, relying heavily on single studies that are limited by methodological constraints such as small sample sizes, cross-sectional designs, and narrow cultural representation. These limitations hinder the generalizability and cumulative understanding of how key psychological constructs operate across broader consumer contexts. Second, the mediating role of TPB constructs—attitude, subjective norm, and PBC—in linking brand-related perceptions to behavioral outcomes remains theoretically promising but empirically fragmented. Despite the intuitive importance of symbolic variables such as brand congruity or scarcity, few studies have systematically evaluated how these variables influence downstream consequences like brand loyalty or word-of-mouth (WoM) behavior. Third, the absence of comprehensive synthesis efforts leaves unresolved questions regarding the robustness, consistency, and boundary conditions of these effects across different market and demographic settings.

In response, this study adopts a meta-analytic methodology to systematically integrate and evaluate findings from a large body of empirical literature. By focusing on three theoretical blocks—antecedents (PS, brand congruity, and PQ), mediating TPB constructs (attitude, SN, PBC, and purchase intention), and behavioral outcomes (brand loyalty and WoM)— this research seeks to quantify the strength and nature of the connections between psychological factors and fast fashion consumption results. By linking consumer psychology with rational behavioral theory, it seeks to establish a comprehensive, theory-grounded framework that not only enriches scholarly discussion but also offers useful advice for brand managers, marketers, and policymakers who are working to connect business success with social and environmental responsibility.

Accordingly, the research pursues three main objectives. First, it evaluates the direct and indirect effects of perceived scarcity, brand congruity, and perceived quality on consumer outcomes through a meta-analytic approach. Second, it examines the mediating roles of the TPB components—attitude, subjective norms, and perceived behavioral control—in explaining how these antecedents influence purchase intention. Finally, it strives to generate a broader and more generalizable understanding of fast fashion consumption patterns while offering implications for both theoretical advancement and practical applications.

## 2. Literature review

### 2.1 Theoretical foundations

This study is grounded in five interrelated theoretical foundations: PS Theory, Self-Congruity Theory, PQ Theory, the TPB, and the Expectation-Confirmation-Loyalty (ECL) Model. Each theory, developed from distinct disciplinary roots—ranging from consumer psychology and marketing to behavioral economics—collectively forms a robust conceptual framework for understanding not only the formation of fast fashion consumption intentions, but also the resulting post-purchase behaviors. This study integrates these theories to examine the consumer decision-making process across both pre-intention and post-intention phases.

The PS theory, initially advanced by Worchel, Lee, and Adewole (1975), argues that when products are perceived to be in limited supply, their desirability increases due to a psychological scarcity effect. This phenomenon has been widely validated across various consumer contexts, from luxury retail to online flash sales. Scarcity can take many forms—such as limited-time availability, exclusive collaborations, or artificial supply constraints—and often evokes emotions such as urgency, anxiety, and Fear of Missing Out (FOMO). In our model, PS functions as a core antecedent variable influencing consumers’ attitudes toward fast fashion brands, their perception of social expectations (SN), and their sense of behavioral control. These, in turn, determine their intention to engage in fast fashion consumption.

Self-Congruity Theory, introduced by Sirgy (1986), is rooted in Rogerian psychology and symbolic interactionism. It emphasizes that consumers prefer brands and products that reflect or reinforce their self-image, including actual, ideal, and social identities. In this framework, brand congruity (CFFB) is a psychological alignment between the consumer’s self-concept and the symbolic meaning of the brand. This alignment deepens emotional involvement and fosters stronger brand attitudes and purchase intentions. Particularly in high-symbolism product categories like fashion, symbolic congruity plays a pivotal role. In this study, CFFB is positioned as a critical antecedent influencing all three TPB constructs and indirectly shaping consumption intention.

The PQ theory, articulated by Zeithaml (1988) and extended by Dodds et al. (1991), frames quality as a subjective perception shaped by product expectations, brand cues, and actual experience. Unlike objective quality metrics, PQ reflects how consumers interpret quality signals—especially in environments like fast fashion where purchases are often impulse-driven and heavily influenced by aesthetics, branding, and digital presentation. In our research model, PQ informs attitudes, SN, and PBC, thereby playing a central role in how consumers form purchase intentions. Digital platforms, influencer marketing, and social proof amplify the influence of PQ, especially among younger, digitally native consumer segments.

The TPB, introduced by Ajzen (1991), continues to be a framework that is very frequently used to explain and forecast how people make decision in a variety of fields. The TPB suggests that behavioral intention is determined by three key factors: a person’s attitude toward the behavior (attitude), the subjective norms they perceive, and their sense of perceived behavioral control. This theory serves as the central mediating framework in our model, linking the psychological and contextual antecedents (PS, CFFB, PQ) to intention (IFFC). In this study, inclusion of TPB allows for a structured understanding of how both rational evaluations and affective responses influence behavioral intentions in fast fashion consumption.

The ECL Model extends the analysis beyond intention to behavioral outcomes. Initially proposed by Oliver (1980) and later refined by Bhattacherjee (2001), the model explains how post-purchase satisfaction and brand loyalty emerge when consumers’ expectations are met or exceeded by actual experience. Our study uses the ECL model to examine how behavioral intention (IFFC) influences downstream loyalty (BL) and WoM. The integration of this model emphasizes that building long-term consumer relationships in fast fashion requires more than stimulating desire—it necessitates consistent delivery on perceived promises.

**Figure 1.**
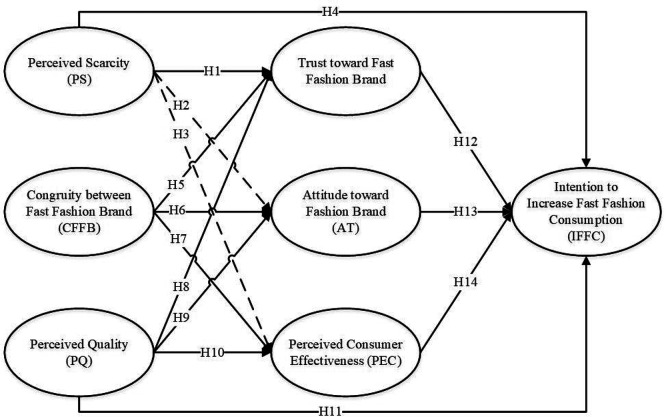
Research model.

In conclusion, each of the five theories contributes a distinct lens: PS, SC, and PQ address different aspects of consumer psychology; TPB provides the behavioral intention mechanism; and the ECL model explains loyalty formation. This multi-theoretical integration offers a comprehensive and layered understanding of fast fashion consumption behavior, combining cognitive, emotional, and contextual dimensions in a single empirical framework (see Figure 1).

### 2.2 Hypotheses development

*2.2.1 The influence of perceived scarcity on attitude, subjective norm, perceived behavioral control, and intention to purchase fast fashion*

PS refers to consumers’ subjective sense that a product is limited in quantity, availability, or subject to purchase constraints ( Chen & Yao, 2018). Brands often deploy scarcity-based marketing tactics—such as limiting the availability or duration of product access—to create a sense of urgency and exclusivity ( Cremer & Loebbecke, 2021; Gupta & Gentry, 2016). These tactics appeal to consumers’ psychological need for ownership, status, and FOMO, all of which have been linked to enhanced consumer engagement and decision-making speed. Attitude ( Ajzen & Fishbein, 2000), a key variable in the TPB, is shaped by both emotional and cognitive factors. When consumers perceive a product as scarce, it often signals desirability and value, leading to more favorable attitudes toward the brand.

Table 1 presents empirical evidence supporting the relationship between PS and consumer attitudes toward fast fashion brands. Across studies conducted in Taiwan, the U.S., China, Turkey, and Indonesia, positive correlations ( *r* ranging from 0.232 to 0.49) were observed between perceived scarcity and favorable brand attitudes. These findings suggest a robust cross-cultural effect. The psychological mechanism behind this relationship can be explained through the Stimulus-Organism-Response (S-O-R) model and TPB, where PS (stimulus) influences internal evaluations (attitude) and results in behavioral intentions. Hence, the following hypothesis is proposed:

H1:
*PS of products positively influences consumers’ attitudes toward fast fashion brands.*

**Table 1.** Summary of Empirical studies on the relationship between PS and attitude toward fast fashion brands.

Author(s) name	Independent variable	Dependent variable	n	r	Method/Country
Chang et al. (2024)	PS	AT	201	0.232	Survey/Taiwan
Cook and Yurchisin (2017)	PS	AT	246	0.49	Survey/USA
Lee et al. (2023)	PS	AT	385	0.252	Survey/China
Köse and Özer Çizer (2021)	PS	AT	399	0.281	Survey/Turkey
Yusuf (2021)	PS	AT	200	0.41	Survey/Indonesia

PS may also affect consumers’ SN, a core component of the TPB that captures an individual’s perception of social expectations to either engage in or abstain from a certain action ( Ajzen, 1991). A consumer who perceives a product to be scarce may experience increased pressure from significant others (family, friends, and influencers) who also value the scarce product. This occurrence can be understood by applying Social Influence Theory ( Cialdini & Goldstein, 2004), which posits that individuals have a greater tendency to adopt the group’s behavior when they perceive group consensus or peer endorsement. Table 2 supports this notion, indicating that PS and SN are moderately and positively correlated when considering the consumption of fast fashion. Based on this evidence, the following hypothesis is proposed:

H2:
*PS of products positively influences consumers’ subjective norms toward fast fashion brands.*

**Table 2.** Summary of Empirical study on the relationship between PS and subjective norm toward fast fashion brands.

Author(s) name	Independent variable	Dependent variable	n	r	Method/Country
Chang et al. (2024)	PS	SN	201	0.35	Survey/Taiwan

The third dimension of TPB, PBC, relates to a person’s judgment on the level of control they have over performing a certain behavior. Interestingly, Meuthia et al. (2023) found an inverse link between PS and PBC; this finding suggests that scarcity may create psychological barriers and feelings of helplessness when consumers believe they lack the ability to access scarce products. This aligns with the principles of the Cognitive Appraisal Theory, where individuals evaluate stressful stimuli based on perceived controllability. As shown in Table 3, if consumers feel that products are overly difficult to obtain, their PBC—and consequently, their purchase intention—may decrease. Accordingly, we propose:

H3:
*PS of products negatively influences consumers’ perceived behavioral control toward fast fashion brands.*

**Table 3.** Summary of Empirical study on the relationship between PS and perceived behavioral control toward fast fashion brands.

Author(s) name	Independent variable	Dependent variable	n	r	Method/Country
Meuthia et al. (2023)	PS	PBC	289	-0.295	Survey/Indonesia

PS has been widely studied as a psychological trigger that influences consumer behavior, particularly in contexts involving limited availability or exclusivity. As shown in Table 4, Chang, Lai, and Yen (2024) found that PS can increase consumers’ willingness to buy by fostering feelings of uniqueness and time-sensitivity for products with restricted availability, which encourages customers to expedite their purchase due to the fear of the item becoming unavailable. Similarly, Cengiz and Şenel (2024) argued that PS increases consumers’ FOMO and impulse-buying tendencies. However, they also emphasized that the effectiveness of scarcity appeals can be highly context-dependent, varying with product category and cultural background. Despite these findings, the connection between PS and the desire to purchase is inconsistent throughout the existing literature. Broeder and Wentink (2022) reported that PS did not significantly induce purchase intentions in their sample, suggesting that the effect may be contingent on additional factors such as consumer characteristics or market conditions. Furthermore, Meuthia et al. (2023) found that PS did not directly or significantly influence panic-driven purchasing habits, which may parallel the dynamics of intention formation in fast fashion consumption. Given these mixed empirical results, the present meta-analysis posits that an overall positive relationship exists between PS and the intent of consumers to engage in greater fast fashion consumption. Accordingly, we propose the following hypothesis:

H4:
*PS is positively associated with consumers’ intention to increase fast fashion consumption.*

**Table 4.** Summary of Empirical study on the relationship between PS and intention to increase fast fashion consumption.

Author(s) name	Independent variable	Dependent variable	n	r	Method/Country
Chang et al. (2024)	PS	IFFC	201	0.195	Survey/Taiwan
Cengiz and Şenel (2024)	PS	IFFC	271	0.663	Survey/Turkey
Yuen et al. (2022)	PS	IFFC	507	0.263	Survey/Singapore
Zhang et al. (2021)	PS	IFFC	509	0.274	Survey/Malaysia
Zhang et al. (2022)	PS	IFFC	488	0.238	Survey/Malaysia
Chen et al. (2022)	PS	IFFC	437	0.155	Survey/China
Omar et al. (2021)	PS	IFFC	157	0.026	Survey/Malaysia
Köse and Özer Çizer (2021)	PS	IFFC	399	0.141	Survey/Turkish
Sung et al. (2021)	PS	IFFC	134	0.1628	Survey/Australia
Broeder and Wentink (2022)	PS	IFFC	208	-0.061	Survey/Netherlands
Meuthia et al. (2023)	PS	IFFC	289	-0.217	Survey/Indonesia

*2.2.2 The effect of congruity on attitude toward fast fashion brands, subjective norm, and perceived behavioral control*

Within branding research, Self-Congruity Theory ( Sirgy, 1982) suggests that individuals tend to prefer brands that reflect or correspond to their self-identity. This alignment—often referred to as brand-self congruity or simply congruity—enhances emotional connection, brand preference, and favorable evaluations. In the fast fashion context, this theory is especially relevant, as consumers are not merely purchasing clothing for functional purposes but are also seeking to express identity, lifestyle, and social affiliation.

Empirical evidence has consistently shown that when a consumer perceives a fast fashion brand to be congruent with their self-image, their attitude toward that brand becomes more favorable. As shown in Table 5, multiple studies across various countries—ranging from the United States to Indonesia, China, and Vietnam—report significant positive correlations between brand congruity and consumer attitudes toward fast fashion brands. For example, Fu et al. (2017) report a strong correlation (r = 0.81) in the U.S., while Setyaningsih & Asnawi (2022) find a similarly high correlation (r = 0.532) in Indonesia.

**Table 5.** Summary of Empirical evidence on the relationship between brand congruity and attitude toward fast fashion brands.

Author(s) name	Independent variable	Dependent variable	n	r	Method/Country
Fu et al. (2017)	CFFB	AT	365	0.81	Survey/USA
Badrinarayanan et al. (2014)	CFFB	AT	316	0.35	Survey/USA
Setyaningsih and Asnawi (2022)	CFFB	AT	204	0.532	Survey/Indonesia
Abimbola et al. (2012)	CFFB	AT	264	0.46	Survey/Australia
Chetioui et al. (2020)	CFFB	AT	610	0.199	Survey/Morocco
Su and Reynolds (2017)	CFFB	AT	420	0.34	Survey/China
Chau and Thanh (2022)	CFFB	AT	510	0.414	Survey/Vietnam
Foroudi et al. (2021)	CFFB	AT	448	0.196	Survey/UK
Lee et al. (2023)	CFFB	AT	385	0.224	Survey/China
Koh et al. (2022)	CFFB	AT	458	0.275	Survey/Korea
Chen et al. (2022)	CFFB	AT	225	0.152	Survey/Taiwan
Shin et al. (2016)	CFFB	AT	854	0.46	Survey/USA
Kang et al. (2012)	CFFB	AT	389	0.455	Survey/USA
Han & Chen (2024)	CFFB	AT	304	0.705	Survey/Thailand
Yen and Mai (2020)	CFFB	AT	539	0.344	Survey/Vietnam

The theoretical underpinning for this relationship is that congruity enhances cognitive consistency, leading to positive affect and reinforcement of self-identity ( Sirgy et al., 2000). Consumers perceive congruent brands as extensions of themselves, which fosters brand loyalty and deepens affective evaluations. Thus, brand managers in the fast fashion industry often focus on tailoring their brand identity to reflect aspirational or socially desirable traits of their target audiences.

H5:
*Congruity between consumer self-image and fast fashion brand identity positively influences consumer attitudes toward the brand.*

Building upon self-congruity theory ( Sirgy, 1982), researchers have explored how congruity between consumer self-image and brand identity extends beyond individual attitudes to influence social perceptions and expectations. As a concept within the TPB ( Ajzen, 1991), subjective norm is defined as the social pressure an individual feels from influential people to either engage in or refrain from a specific behavior. In the context of fast fashion, if a consumer perceives a brand to align closely with their identity and values, this congruity may enhance their sensitivity to others’ opinions about the brand and increase their conformity to perceived social expectations.

The empirical evidence summarized in Table 6 shows a significant and consistent link between the alignment of a brand with a brand congruity and SN. The findings show that when consumers feel a fashion brand is aligned with their sense of self-image, they have a greater tendency to expect that significant others, like friends and influencers, would endorse their choice of the brand. This alignment fosters a normative belief that strengthens behavioral intentions to engage in consumption aligned with group expectations.

**Table 6.** Summary of Empirical evidence on the relationship between brand congruity and subjective norms toward fast fashion brands.

Author(s) name	Independent variable	Dependent variable	n	r	Method/Country
Chang et al. (2024)	CFFB	SN	201	0.295	Survey/Taiwan
Setyaningsih and Asnawi (2022)	CFFB	SN	204	0.337	Survey/Indonesia
Lee et al. (2023)	CFFB	SN	385	0.251	Survey/China
Chau and Thanh (2022)	CFFB	SN	510	0.332	Survey/Vietnam
Chen et al. (2022)	CFFB	SN	225	0.212	Survey/Taiwan

Additionally, this relationship is framed by social identity theory ( Tajfel & Turner, 1981), which states that people’s identity is partly a result of their connection to social groups. As such, fast fashion brands that reflect collective or group-based identities (e.g., youth culture, sustainability-conscious groups) may reinforce both individual and normative motivations to engage with the brand.

H6:
*Congruity between consumer self-image and fast fashion brand identity positively influences subjective norms toward the brand.*

According to Ajzen’s (1991) TPB, PBC is the degree to which an individual feels that performing a certain action would be either easy or difficult. It shares conceptual overlap with Bandura’s notion of self-efficacy and reflects one’s belief in their capacity to act despite potential barriers or facilitators. Recent research has increasingly integrated self-congruity theory ( Sirgy, 1982) into the TPB framework, emphasizing that when a brand’s image aligns with a consumer’s self-concept, it can impact not only their attitudes and subjective norms but also their sense of control.

Empirical findings, as summarized in Table 7, provide strong support for this proposition. Studies across diverse contexts—including Vietnam ( Chau & Thanh, 2022), the U.S. ( Shin et al., 2016), and Thailand ( Han & Chen, 2024)—have reported positive and significant correlations between brand congruity and PBC. These results suggest that when fast fashion brands resonate with a consumer’s self-identity, they foster a sense of agency, making the act of purchasing feel more feasible, appropriate, and achievable.

**Table 7.** Summary of Empirical evidence on the relationship between brand congruity and perceived behavioral control toward fast fashion brands.

Author(s) name	Independent variable	Dependent variable	n	r	Method/Country
Chau and Thanh (2022)	CFFB	PBC	510	0.372	Survey/Vietnam
Shin et al. (2016)	CFFB	PBC	854	0.43	Survey/USA
Han and Chen (2024)	CFFB	PBC	304	0.402	Survey/Thailand
Yen and Mai (2020)	CFFB	PBC	539	0.55	Survey/Vietnam

This theoretical integration is especially relevant in fast fashion, where brand symbolism plays a critical role. Products are not merely functional; they serve as social markers of identity and status. Thus, when a consumer sees a brand as an extension of themselves, the psychological barriers to purchase are lowered. The increased confidence and fluency in decision-making fostered by congruity reinforce one’s perception of behavioral control, enhancing overall purchase intention and consumer empowerment.

H7:
*Congruity between consumer self-image and fast fashion brand identity positively influences perceived behavioral control.*

*2.2.3 Perceived quality positively influences consumers’ attitude toward fast fashion brands, subjective norm, perceived behavioral control, and intention to purchase fast fashion*

Perceived Quality is a central concept in consumer behavior research. According to Zeithaml (1988), it reflects a consumer’s assessment of a product’s overall excellence. These perceptions are critical in attitude-behavior and expectancy-value frameworks, as they affect both emotional responses and evaluations of the brand, ultimately influencing consumer attitudes. Attitude, in turn, is conceptualized as a consumer’s overall evaluative disposition—favorable or unfavorable—toward engaging with a particular brand or product category ( Ajzen, 1991). When consumers perceive a brand’s offerings as being of high quality—whether in terms of aesthetics, material durability, functionality, or value— it becomes more probable that they will have a positive view of the brand.

Empirical studies as shown in Table 8 have consistently demonstrated that PQ positively influences brand attitude. Gelaidan et al. (2023) demonstrated that perceived service quality plays a significant role in shaping consumer attitudes toward sustainable metro services in the transportation sector. Likewise, in the context of organic foods, factors such as taste and safety have been found to enhance consumer assessments and intentions to buy ( Teixeira et al., 2021). Within the beauty and personal care industry, Echchad and Ghaith (2022) found that product quality and environmental values jointly contributed to a favorable attitude toward green cosmetics. In service-oriented contexts, Foroudi et al. (2021) highlighted the effect of high-quality perception on consumers’ attitude toward restaurant brands, and similar effects were documented in ESG-related brand perception studies ( Koh et al., 2022). Notably, Wang et al. (2020) identified that PQ was a key determinant of Chinese consumers’ attitudes toward certified pork, suggesting that this construct may hold cross-industry and cross-cultural relevance.

**Table 8.** Summary of Empirical evidence on the relationship between perceived quality and attitude toward fast fashion brand.

Author(s) name	Independent variable	Dependent variable	n	r	Method/Country
Koh et al. (2022)	PQ	AT	458	0.378	Survey/Korean
Foroudi et al. (2021)	PQ	AT	448	0.323	Survey/UK
Gelaidan et al. (2023)	PQ	AT	1334	0.207	Survey/Qatar
Hou et al. (2021)	PQ	AT	503	0.71	Survey/China
AlHadid et al. (2022)	PQ	AT	442	0.299	Survey/Jordan
Wang et al. (2020)	PQ	AT	844	0.545	Survey/Hong Kong
Teixeira et al. (2021)	PQ	AT	206	0.85	Survey/Portugal
Echchad and Ghaith (2022)	PQ	AT	204	0.31	Survey/Hungary
Jalil and Shaharuddin (2020)	PQ	AT	98	0.64	Survey/Malaysia

In the fast fashion context, although relatively underexplored, the positive link between PQ and consumer attitude is theoretically transferable. Fast fashion brands typically compete by offering trend-responsive, affordable clothing at scale. If consumers perceive these products as being well-designed, comfortable, or offering good value for money, such perceptions are likely to translate into favorable brand evaluations. As evidenced by studies from Koh et al. (2022), Hou et al. (2021), and Teixeira et al. (2021), fast fashion consumers in various markets tend to report positive attitudes when they associate the brand with reliability, style, and price-quality alignment.

Therefore, grounded in both theoretical expectations and cross-sector empirical findings, this study proposes the following hypothesis:

H8:
*PQ positively influences consumers’ attitude toward fast fashion brands and perceived behavioral control toward fast fashion brands.*

While the role of PQ in shaping attitudes and purchase intentions is well-documented, its impact on subjective norm—a key construct in the TPB—has received limited attention, particularly in fast fashion. Subjective norm captures the perceived expectations of important others, including friends, family, or peers, about engaging in specific behaviors ( Ajzen, 1991). Emerging studies suggest that perceptions of brand quality can influence consumers’ own evaluations as well as their beliefs regarding what behaviors are socially endorsed among peers.

Empirical studies as shown in Table 9 provide emerging support for this association. For instance, Nurhidayat et al. (2023) showed a strong correlation between PQ and subjective norms in the Indonesian fashion context (r = 0.645), which suggests that when consumers view a fashion brand as high-quality, it becomes more probable that they will feel others approve of using the brand as well. Similarly, research in China and Hong Kong ( Hou et al., 2021; Wang et al., 2020) confirmed that PQ exerts a substantial effect on subjective norm, indicating a shared valuation process where quality signals influence collective norms of acceptability and desirability.

**Table 9.** Summary of Empirical evidence on the relationship between perceived quality and subjective norm toward fashion brand.

Author(s) name	Independent variable	Dependent variable	n	r	Method/Country
Nurhidayat et al. (2023)	PQ	SN	100	0.645	Survey/Indonesia
Hou et al. (2021)	PQ	SN	503	0.57	Survey/China
Wang et al. (2020)	PQ	SN	844	0.306	Survey/Hong Kong

The mechanism underlying this relationship may stem from social validation: consumers tend to internalize quality cues not only as personal assessments but also as indicators of how others might evaluate the brand. In fast fashion, where peer identity and trend alignment are salient, a high PQ can serve as a normative anchor that guides social behavior. That is, consumers may perceive that others expect them to use or recommend brands that are perceived as reliable, stylish, or environmentally responsible—attributes commonly associated with quality.

Furthermore, the symbolic value attached to quality in fashion contexts—especially in cultures with strong collectivist orientations—amplifies the likelihood that quality perceptions translate into social conformity pressures. Accordingly, it is proposed that when consumers view a fast fashion brand as high-quality, they are more inclined to think that important referents would approve of their purchasing decisions.

Based on this reasoning, the following hypothesis is proposed:

H9:
*PQ positively influences consumers’ subjective norm toward fast fashion brands.*

PBC, a central construct in the TPB, reflects an individual’s perception of the ease or difficulty of performing a given behavior, often influenced by both internal factors (e.g., skills, knowledge) and external resources (e.g., time, money, support) ( Ajzen, 1991). While PBC is traditionally conceptualized as distinct from attitudinal or normative beliefs, emerging research has suggested that external cues—such as perceived product quality—may indirectly shape individuals’ sense of behavioral control, especially in consumption contexts where quality is linked to trust, predictability, and satisfaction.

Although the relationship between PQ and PBC has not been widely examined in fast fashion specifically, studies as shown in Table 10 offer theoretical support for a positive link. Wang et al. (2020) found that in the context of certified pork consumption in China, perceived product quality significantly enhanced subjective norm and interacted positively with PBC, indicating that higher quality perception may enhance consumers’ confidence in engaging in the intended behavior. Similarly, Hou et al. (2021) demonstrated that perceived train service quality significantly strengthened PBC in a high-speed rail setting, as better service experiences led passengers to feel more capable of using the service reliably and regularly.

**Table 10.** Summary of Empirical Evidence on the Relationship between Perceived Quality and Subjective Norm toward Fashion Brand.

Author(s) name	Independent variable	Dependent variable	n	r	Method/Country
Gelaidan et al. (2023)	PQ	PBC	1334	0.417	Survey/Qatar
Hou et al. (2021)	PQ	PBC	503	0.54	Survey/China
Abu-Taieh et al. (2022)	PQ	PBC	403	0.154	Survey/Jordan
Wang (2020)	PQ	PBC	844	0.442	Survey/Hong Kong
Yu et al. (2024)	PQ	PBC	1109	-0.07	Survey/China

Further evidence from Gelaidan et al. (2023), Abu-Taieh et al. (2022), and Yu et al. (2024) shows mixed but generally positive correlations between PQ and PBC in the context of fashion and services. These findings support the idea that when consumers believe a product or service is of high quality, they are more likely to feel in control of their decision to use or purchase it—due to expectations of reliability, ease of use, and lower risk of dissatisfaction.

Applying these insights to the fast fashion domain, we can infer that consumers who perceive fast fashion brands as high-quality may also feel more confident in their ability to make satisfying and socially supported purchase decisions. High PQ may reduce perceived risks, increase perceived access, and foster a sense of empowerment in the purchase process—thereby strengthening perceived behavioral control.

Therefore, the following hypothesis is proposed:

H10:
*PQ positively influences consumers’ perceived behavioral control toward fast fashion brands.*

For a long time, PQ has been considered a fundamental influence on consumer choices, playing a significant role in forming behavioral intentions in various consumption contexts. In the framework of the TPB, PQ is often incorporated as an external belief-based factor that indirectly or directly influences key outcome variables, such as purchase intention or behavioral control. Although the influence of perceived quality (PQ) on fast fashion consumption intention has received limited scholarly attention, emerging empirical findings indicate a strong positive association between the two variables (see Table 11).

**Table 11.** Summary of Empirical evidence on the relationship between perceived quality and intention to increase fast fashion consumption.

Author(s) name	Independent variable	Dependent variable	n	r	Method/Country
Aquinia et al. (2021)	PQ	IFFC	100	0.202	Survey/Indonesia
Islam et al. (2022)	PQ	IFFC	236	0.207	Survey/Bangladesh
Akem and Cheumar (2024)	PQ	IFFC	381	0.164	Survey/Malaysia
Filieri and Lin (2017)	PQ	IFFC	321	0.38	Survey/UK
Nurhidayat et al. (2023)	PQ	IFFC	100	0.22	Survey/Indonesia
Das (2014)	PQ	IFFC	365	0.496	Survey/Malaysia
Wang et al. (2020)	PQ	IFFC	844	0.318	Survey/Hong Kong
Kanwar and Huang (2022)	PQ	IFFC	400	0.71	Survey/Taiwan
Hardiyanto et al. (2020)	PQ	IFFC	210	0.731	Survey/Indonesia
Cayaban et al. (2023)	PQ	IFFC	407	0.109	Survey/Philippines
Hou et al. (2021)	PQ	IFFC	503	0.28	Survey/China
Abu-Taieh et al. (2022)	PQ	IFFC	403	0.164	Survey/Jordan

As an example, Wang et al. (2020) showed that Chinese consumers who perceived higher product quality in certified pork exhibited stronger intentions to purchase, mediated by enhanced confidence and PBC. Similarly, in the high-speed rail context, Hou et al. (2021) found that a high standard of service quality significantly influenced both a person’s sense of behavioral control and their intentions. In the public transportation domain, Gelaidan et al. (2023) confirmed that service quality enhances users’ perceived ability and motivation to engage in the intended behavior. Although these studies are drawn from non-fashion contexts, they consistently support the view that high PQ contributes to increased confidence, perceived control, and ultimately stronger behavioral intentions.

Drawing from these findings, recent research in the fast fashion industry has increasingly confirmed a direct effect of Perceived Quality (PQ) on consumers’ purchase intentions. Studies from diverse countries—including Indonesia ( Aquinia et al., 2021; Hardiyanto et al., 2020), Malaysia ( Akem & Cheumar, 2024), China ( Hou et al., 2021), and the UK ( Filieri & Lin, 2017)—report moderate to strong positive correlations (ranging from r = 0.164 to r = 0.731) between consumers’ perception of product quality and their intention to increase consumption. These results suggest that consumers who perceive fast fashion products as stylish, well-made, and reasonably priced are more likely to express intentions to purchase more frequently or in larger quantities.

In fast fashion, where purchase decisions are often spontaneous and influenced by aesthetic appeal, PQ may serve not only as a signal of product value but also as a justification for repeat or increased purchases. Higher quality perception reduces cognitive dissonance, elevates satisfaction, and enhances consumers’ willingness to re-engage with the brand. Thus, when consumers believe that fast fashion products meet their expectations in terms of design, durability, and affordability, their intention to increase consumption is likely to be strengthened.

Based on these insights, the following hypothesis is proposed:

H11:
*PQ positively influences consumers’ intention to increase fast fashion consumption.*

*2.2.4 The effect of subjective norms and perceived behavioral control on intentions to increase fast fashion consumption*

In the TPB model, PBC is a critical determinant of behavioral intention. It is defined as an individual’s sense of their capacity to perform a specific behavior, influenced by both internal elements (e.g., confidence, skills) and external factors (e.g., time, money, social support) as described by Ajzen (1991). In consumer research, PBC has been consistently linked to purchase intentions, particularly in contexts where decision-making involves autonomy, convenience, or perceived accessibility.

In the fast fashion sector, where product availability is high and purchasing decisions are often impulsive yet frequent, PBC plays a particularly salient role. When consumers feel they have sufficient control—such as financial affordability, access to retail channels, and confidence in personal style or product selection—they are more likely to act on their consumption impulses. Prior empirical studies have confirmed this relationship in adjacent domains. As shown in Table 12, Gelaidan et al. (2023) and Hou et al. (2021) reported that higher PBC significantly enhanced behavioral intentions in public transportation and service settings. Similarly, Wang et al. (2020) found that Chinese consumers’ PBC was positively correlated with their intention to purchase certified pork, especially when quality and availability were perceived as favorable.

**Table 12.** Summary of Empirical evidence on the relationship between attitude toward fast fashion brand and intention to increase fast fashion consumption.

Author(s) name	Independent variable	Dependent variable	n	r	Method/Country
Chang et al. (2024)	AT	IFFC	201	0.434	Survey/Taiwan
Zhu et al. (2024)	AT	IFFC	913	0.353	Survey/China
Nguyen et al. (2022)	AT	IFFC	638	0.405	Survey/Vietnam
Chaudhary and Bisai (2018)	AT	IFFC	202	0.4	Survey/India
Cayaban et al. (2023)	AT	IFFC	407	0.656	Survey/Indonesia
Koh et al. (2022)	AT	IFFC	458	0.854	Survey/Korean
Rostiani and Kuron (2019)	AT	IFFC	336	0.41	Survey/Indonesia
Hageman et al. (2024)	AT	IFFC	155	0.606	Survey/UK
Kashif et al. (2018)	AT	IFFC	484	0.31	Survey/Pakistan
Chen et al. (2022)	AT	IFFC	225	0.662	Survey/Taiwan
Badrinarayanan et al. (2014)	AT	IFFC	316	0.57	Survey/USA
Yusuf (2021)	AT	IFFC	200	0.511	Survey/Indonesia
Harjadi and Gunardi (2022)	AT	IFFC	419	0.314	Survey/Indonesia
Aziz (2019)	AT	IFFC	319	0.308	Survey/Pakistan
Phang and Ilham (2023)	AT	IFFC	394	0.449	Survey/Malaya
Rao et al. (2022)	AT	IFFC	345	0.245	Survey/USA
Chetioui et al. (2020)	AT	IFFC	610	0.462	Survey/Morocco
Stolz (2022)	AT	IFFC	469	0.172	Survey/Germany
Roh et al. (2022)	AT	IFFC	251	0.486	Survey/Korea
Li et al. (2022)	AT	IFFC	389	0.646	Survey/Malaysia
Hao and Jusoh (2024)	AT	IFFC	211	0.872	Survey/Malaysia
Nam et al. (2017)	AT	IFFC	542	0.4	Survey/USA
Suha and Chaichi (2018)	AT	IFFC	240	0.512	Survey/Malaysia
Chetioui et al. (2021)	AT	IFFC	298	0.55	Survey/Moroccan
Rafiq et al. (2020)	AT	IFFC	452	0.731	Survey/
Lili et al. (2022)	AT	IFFC	301	0.393	Survey/Malaysia
Ma et al. (2023)	AT	IFFC	471	0.213	Survey/Vietnam
Gelaidan et al. (2023)	AT	IFFC	1334	0.783	Survey/Qatar
Islam et al. (2022)	AT	IFFC	236	0.578	Survey/Bangladesh
Teixeira et al. (2021)	AT	IFFC	206	0.92	Survey/Portugal
Echchad and Ghaith (2022)	AT	IFFC	204	0.786	Survey/Hungary
Köse and Özer Çizer (2021)	AT	IFFC	399	0.687	Survey/Turkish
Jain (2020)	AT	IFFC	215	0.143	Survey/India
Yang and Ahn (2020)	AT	IFFC	456	0.733	Survey/South korea
Hasan and Suciarto (2020)	AT	IFFC	100	0.527	Survey/Indonesia
Liu et al. (2020)	AT	IFFC	485	0.21	Survey/China
Bhati et al. (2022)	AT	IFFC	449	0.359	Survey/India
Lee et al. (2023)	AT	IFFC	385	0.489	Survey/China
Setyaningsih and Asnawi (2022)	AT	IFFC	204	0.718	Survey/Indonesia
Hou et al. (2021)	AT	IFFC	503	0.67	Survey/China
AlHadid et al. (2022)	AT	IFFC	442	0.998	Survey/Jordan
Wang et al. (2020)	AT	IFFC	844	0.731	Survey/Hong Kong
Jalil and Shaharuddin (2020)	AT	IFFC	98	0.83	Survey/Malaysia
Shin et al. (2016)	AT	IFFC	854	0.44	Survey/USA

Translating this to fast fashion, consumers who believe they can easily afford, access, and choose fashion products that match their preferences are more inclined to increase consumption frequency or quantity. PBC may be shaped by factors such as the affordability of fast fashion items, the ubiquity of both physical and online retail platforms, and consumers’ confidence in assembling fashionable outfits. This sense of control reduces psychological barriers and heightens the likelihood of engaging in repeat consumption behavior. Thus, consistent with TPB and empirical evidence across multiple consumption contexts, this study proposes the following hypothesis:

H12:
*Perceived behavioral control positively influences consumers’ intention to increase fast fashion consumption.*

In the original TPB, PBC is considered to directly influence behavioral intention, alongside the effects of attitude and subjective norms. However, recent extensions of TPB and related consumer behavior models suggest that PBC may also indirectly shape other cognitive-affective antecedents, particularly consumer attitude. Attitude is a consumer’s total evaluation, either positive or negative, regarding their participation in a certain behavior or their support for a brand ( Ajzen, 1991). When consumers feel they have control over their actions, including the ease, competence, and resources needed to make decisions, this increases the likelihood of generating positive judgments toward the brand or behavior.

Empirical studies as shown in Table 13 support this expanded conceptual pathway. For example, in the context of green consumption, consumers who perceive fewer barriers to purchasing eco-friendly products tend to develop more favorable attitudes toward those products ( Yadav & Pathak, 2017). In online shopping environments, users with greater perceived control over navigation, purchase steps, or platform familiarity report more positive evaluations of the shopping experience ( Pappas, 2016). These findings indicate that a sense of behavioral control can reduce anxiety, increase self-efficacy, and enhance trust—factors that are foundational to favorable attitude formation.

**Table 13.** Summary of Empirical evidence on the relationship between subjective norm toward fashion brand and intention to increase fast fashion consumption.

Author(s) name	Independent variable	Dependent variable	n	r	Method/Country
Chang et al. (2024)	SN	IFFC	201	0.18	Survey/Taiwan
Zhu et al. (2024)	SN	IFFC	913	0.152	Survey/China
Nguyen et al. (2022)	SN	IFFC	638	0.208	Survey/Vietnam
Chaudhary and Bisai (2018)	SN	IFFC	202	-0.02	Survey/India
Nurhidayat et al. (2023)	SN	IFFC	100	0.258	Survey/Indonesia
Cayaban et al. (2023)	SN	IFFC	407	0.074	Survey/Indonesia
Rostiani and Kuron (2019)	SN	IFFC	336	0.18	Survey/Indonesia
Kashif et al. (2018)	SN	IFFC	484	0.092	Survey/Pakistan
Yusuf (2021)	SN	IFFC	200	0.266	Survey/Indonesia
Harjadi and Gunardi (2022)	SN	IFFC	419	0.810	Survey/Indonesia
Aziz (2019)	SN	IFFC	319	0.238	Survey/Pakistan
Phang and Ilham (2023)	SN	IFFC	394	0.428	Survey/Malaya
Rao et al. (2022)	SN	IFFC	345	0.293	Survey/USA
Stolz (2022)	SN	IFFC	469	0.228	Survey/Germany
Roh et al. (2022)	SN	IFFC	251	0.332	Survey/Korea
Li et al. (2022)	SN	IFFC	389	0.52	Survey/Malaysia
Hao and Jusoh (2024)	SN	IFFC	211	0.771	Survey/
Nam et al. (2017)	SN	IFFC	542	0.32	Survey/USA
Suha and Chaichi (2018)	SN	IFFC	240	0.442	Survey/Malaysia
Islam et al. (2022)	SN	IFFC	236	0.12	Survey/Bangladesh
Echchad and Ghaith (2022)	SN	IFFC	204	-0.081	Survey/Hungary
Akem and Cheumar (2024)	SN	IFFC	381	-0.037	Survey/Malaysia
Filieri and Lin (2017)	SN	IFFC	321	0.169	Survey/UK
Teixeira et al. (2021)	SN	IFFC	206	0.13	Survey/Portugal
Jain (2020)	SN	IFFC	215	0.257	Survey/India
Yang and Ahn (2020)	SN	IFFC	456	0.329	Survey/South korea
Hasan and Suciarto (2020)	SN	IFFC	100	-0.28	Survey/Indonesia
Liu et al. (2020)	SN	IFFC	485	0.13	Survey/China
Bhati et al. (2022)	SN	IFFC	449	0.337	Survey/India
Hou et al. (2021)	SN	IFFC	503	0.42	Survey/China
Wang et al. (2020)	SN	IFFC	844	0.317	Survey/Hong Kong
Jalil and Shaharuddin (2020)	SN	IFFC	98	0.11	Survey/Malaysia
Chetioui et al. (2020)	SN	IFFC	610	0.141	Survey/Morocco
Shin et al. (2016)	SN	IFFC	854	0.05	Survey/USA

Applied to fast fashion, this relationship is particularly relevant. When consumers believe they have the autonomy to choose affordable, trendy items and can easily access and evaluate product information, their perceived control reinforces confidence and satisfaction. This positive user experience, in turn, fosters a favorable affective evaluation of the brand. In contrast, if consumers feel constrained—by price, sizing availability, or ethical concerns—their attitude may be tempered regardless of the product offering.

Therefore, in line with the extended TPB and supporting empirical evidence, we propose the following hypothesis:

H13:
*Perceived behavioral control positively influences consumers’ attitude toward fast fashion brands.*

In the TPB, Ajzen (1991) defines PBC as one’s assessment of their ability to perform a given action, shaped by resources, opportunities, and constraints. While PBC is generally considered a robust predictor of behavioral intention, its influence on fast fashion consumption remains empirically inconsistent across contexts and cultures.

The empirical studies as shown in Table 14 report a significant and positive relationship between PBC and the intention to increase consumption. For example, Cayaban et al. (2023) discovered that Filipino consumers with confidence in their ability to find and afford a wide range of fashion items were more prone to have a stronger intent to consume. The availability of product choices and the convenience of access were highlighted as core dimensions enhancing PBC. Likewise, Liu et al. (2023) and Zhu et al. (2024) in Korea and China, respectively, observed strong correlations (r = 0.58 to 0.787) between PBC and fast fashion consumption intention, indicating that perceived ease in navigating retail environments plays a crucial role in fostering behavioral intention.

**Table 14.** Summary of Empirical evidence on the relationship between perceived behavioral control toward fashion brand and intention to increase fast fashion consumption.

Author(s) name	Independent variable	Dependent variable	n	r	Method/Country
Chang et al. (2024)	PBC	IFFC	201	0.207	Survey/Taiwan
Zhu et al. (2024)	PBC	IFFC	913	0.58	Survey/China
Liu et al. (2023)	PBC	IFFC	400	0.787	Survey/Korea
Chaudhary and Bisai (2018)	PBC	IFFC	202	0.74	Survey/India
Cayaban et al. (2023)	PBC	IFFC	407	0.214	Survey/Indonesia
Rostiani and Kuron (2019)	PBC	IFFC	336	0.099	Survey/Indonesia
Kashif et al. (2018)	PBC	IFFC	484	0.766	Survey/Pakistan
Aziz (2019)	PBC	IFFC	319	0.942	Survey/Pakistan
Phang and Ilham (2023)	PBC	IFFC	394	0.236	Survey/Malaya
Rao et al. (2022)	PBC	IFFC	345	0.249	Survey/USA
Stolz (2022)	PBC	IFFC	469	0.247	Survey/Germany
Li et al. (2022)	PBC	IFFC	389	0.423	Survey/Malaysia
Giantari et al. (2013)	PBC	IFFC	150	0.602	Survey/Indonesia
Hao and Jusoh (2024)	PBC	IFFC	211	0.766	Survey/Malaysia
Nam et al. (2017)	PBC	IFFC	542	0.06	Survey/USA
Gelaidan et al. (2023)	PBC	IFFC	1334	0.175	Survey/Qatar
Islam et al. (2022)	PBC	IFFC	236	0.184	Survey/Bangladesh
Nurhidayat et al. (2023)	PBC	IFFC	100	0.049	Survey/Indonesia
Teixeira et al. (2021)	PBC	IFFC	206	0.08	Survey/Portugal
Jain (2020)	PBC	IFFC	215	0.272	Survey/India
Hasan and Suciarto (2020)	PBC	IFFC	100	0.572	Survey/Indonesia
Liu et al. (2020)	PBC	IFFC	485	0.1	Survey/China
Bhati et al. (2022)	PBC	IFFC	449	0.316	Survey/India
Hou et al. (2021)	PBC	IFFC	503	0.22	Survey/China
Wang et al. (2020)	PBC	IFFC	844	0.57	Survey/Hong Kong
Yusuf (2021)	PBC	IFFC	200	-0.003	Survey/Indonesia
Harjadi and Gunardi (2022)	PBC	IFFC	419	-0.09	Survey/Indonesia
Chetioui et al. (2020)	PBC	IFFC	610	0.193	Survey/Morocco
Ma et al. (2023)	PBC	IFFC	471	0.088	Survey/Vietnam
Shin et al. (2016)	PBC	IFFC	854	0.34	Survey/USA

Some studies have produced conflicting results. Yusuf (2021) reported that PBC did not significantly influence behavioral intention in online fashion shopping, which he explained by consumers’ sense of limited control in digital purchase settings. Similarly, Nurhidayat et al. (2023) observed weak or insignificant effects of PBC in housing contexts, potentially due to respondents’ limited decision-making experience.

Looking beyond fashion, PBC has been found to influence behavioral intentions in domains such as tourism real estate ( Ma et al., 2023), organic food ( Boobalan & Nachimuthu, 2020), and post-pandemic retail consumption ( Liu et al., 2023). These findings emphasize the contextual sensitivity of PBC, suggesting that its predictive power may depend on product familiarity, cost structure, and perceived risk. For instance, in high-involvement product categories such as real estate or housing, the effect of PBC may be muted by structural barriers like affordability or bureaucratic constraints. In contrast, low-involvement categories like fast fashion may provide more opportunity for PBC to shape consumer intention.

Given this variation, it is premature to generalize PBC’s effectiveness across all consumption settings. Yet, the cumulative evidence indicates that in fast fashion—characterized by low entry barriers, trend responsiveness, and wide accessibility—consumers who feel empowered in their decision-making processes are more inclined to increase their purchase behavior. Accordingly, this study proposes the following hypothesis:

H14:
*Perceived behavioral control positively influences consumers’ intention to increase fast fashion consumption.*

*2.2.5 The effect of intentions to increase on brand loyalty toward fast fashion brand*

Brand loyalty refers to a consumer’s ongoing preference for and commitment to a particular brand over time. This loyalty is often demonstrated through repeated purchases, emotional attachment, and a readiness to tolerate minor product flaws or price differences in favor of the favored brand. In marketing theory, loyalty is not only a key indicator of long-term brand performance but also a crucial mediator between consumer satisfaction and firm profitability. While existing empirical studies have seldom focused specifically on the fast fashion sector, considerable evidence from related fields suggests that purchase intention plays a pivotal role in cultivating brand loyalty.

Purchase intention, often described as the consumer’s expressed probability or will to buy a product in the next time, is typically regarded as an antecedent of both actual purchasing behavior and attitudinal commitment ( Yasin & Shamim, 2013; Das, 2014). Consumers who report a high intention to increase their consumption of a brand’s offerings typically exhibit underlying positive evaluations of that brand—whether based on PQ, emotional resonance, brand symbolism, or utility value. These evaluations, when reinforced over time, foster the emergence of brand loyalty.

Numerous studies as shown in Table 15 support this causal chain. Das (2014), in the Indian non-food retail context, demonstrated that purchase intention was significantly associated with loyalty, particularly when driven by utilitarian and hedonic value. Similarly, Laksamana (2018) examined Indonesian banking consumers and found that purchase intention strongly predicted brand loyalty, suggesting the robustness of this relationship across diverse service industries. In the green marketing domain, Panda et al. (2020) highlighted that purchase intention driven by environmental concern translated into repeat purchases and brand advocacy. Pangaribuan et al. (2020), examining Indonesia’s Luwak White Coffee, confirmed that repeated behavioral intention was one of the most effective predictors of long-term loyalty.

**Table 15.** Summary of Empirical evidence on the relationship between intention to increase fast fashion consumption and brand loyalty toward fast fashion brand.

Author(s) name	Independent variable	Dependent variable	n	r	Method/Country
Laksamana (2018)	IFFC	BL	286	0.565	Survey/Indonesia
Panda et al. (2020)	IFFC	BL	331	0.475	Survey/India
Pangaribuan et al. (2020)	IFFC	BL	100	0.425	Survey/Indonesia
Mookda et al. (2020)	IFFC	BL	351	0.47	Survey/Malaysia
Panda et al. (2020)	IFFC	BL	331	0.475	Survey/UK
Das (2014)	IFFC	BL	365	0.367	Survey/India

Although fast fashion is often associated with low involvement and impulsivity, these traits do not necessarily preclude brand loyalty formation. Fast fashion brands like Zara, H&M, or Uniqlo can evoke strong consumer attachment through brand image consistency, product availability, and lifestyle alignment. A consumer who repeatedly intends to shop from the same fast fashion brand—driven by satisfaction, habit, or convenience—may develop both behavioral and attitudinal loyalty over time.

Moreover, the psychological mechanisms linking intention and loyalty are reinforced through positive reinforcement cycles. The more frequently a consumer acts on their purchase intention and experiences satisfaction, the more likely they are to internalize a loyal mindset toward the brand. This loyalty is expressed through increased retention, greater tolerance for product shortcomings, and stronger emotional endorsement.

Drawing upon theoretical principles and research findings from similar domains, this study proposes the following hypothesis:

H15:
*Intention to increase fast fashion consumption positively influences consumers’ loyalty toward fast fashion brands.*

*2.2.6 The effect of intention to increase fast fashion consumption on WoM behavior*

WoM behavior, both offline and online, is a crucial manifestation of post-consumption engagement that shapes brand perception and market diffusion. In the age of digital consumerism and social sharing, particularly in visually-driven industries like fashion, WoM serves as a vital conduit for brand influence, social validation, and peer persuasion. While few empirical studies currently explore this relationship in the fast fashion context, a substantial body of literature from various industries consistently supports a positive relationship between purchase intention and WoM behavior.

Purchase intention reflects a consumer’s motivational state to engage in buying behavior and is frequently cited as a predictor of not only actual purchase but also related behaviors such as recommending, reviewing, and advocating for a brand ( Yasin & Shamim, 2013; Abu-Taieh et al., 2022). When a consumer develops a strong intention to purchase, this mental commitment often spills over into interpersonal communication, particularly when the brand aligns with their identity or delivers a satisfying experience.

In empirical research as shown in Table 16, Yasin and Shamim (2013) observed that mobile phone users with high purchase intentions were significantly more likely to engage in WoM activities. This behavior was reinforced by emotional involvement and social influence. In a service context, Abu-Taieh et al. (2022) reported similar findings among mobile banking consumers in Jordan, where behavioral intention translated into active advocacy and brand promotion. Most notably, Farzin et al. (2023) showed that in the eco-fashion space, consumers’ purchase intentions had a direct and significant effect on electronic WoM suggesting that intention-driven communication behavior is not confined to functional or utilitarian products but extends to fashion as well.

**Table 16.** Summary of Empirical evidence on the relationship between intention to increase fast fashion consumption and word-of- mouth toward fashion brand.

Author(s) name	Independent variable	Dependent variable	n	r	Method/Country
Yasin and Shamim (2013)	IFFC	WoM	265	0.318	Survey/Pakistan
Abu-Taieh et al. (2022)	IFFC	WoM	403	0.746	Survey/Jordan
Farzin et al. (2023)	IFFC	WoM	389	0.917	Survey/Iran
Shah et al. (2020)	IFFC	WoM	307	0.236	Survey/China
Jo (2023)	IFFC	WoM	645	0.525	Survey/Korea

Fast fashion’s dynamic and trend-sensitive ecosystem further amplifies the potential for intention to drive WoM. Consumers are often highly engaged with their fashion choices and motivated to share outfit inspirations, product hauls, and brand experiences across social media platforms. A strong intention to consume not only increases the likelihood of purchase but also enhances the consumer’s willingness to share their enthusiasm with peers—especially in youth-driven or style-conscious segments. In this way, WoM becomes a behavioral extension of consumption intention.

Furthermore, WoM is shaped by motivational factors such as self-enhancement, social affiliation, and informational utility. When a consumer intends to make a purchase, they are often gathering or disseminating information, which naturally involves discussing the brand with others. This process is especially relevant in fast fashion, where new styles, limited-time collections, and social comparison cues heighten the likelihood of shared conversations.

In sum, although fast fashion-specific research is limited, the consistent empirical pattern across retail, service, and fashion-related studies provides strong justification for the following hypothesis:

H16:
*Intention to increase fast fashion consumption positively influences consumers’ WoM behavior toward fast fashion brands.*

## 3. Methodology

### 3.1 Meta-analysis

Meta-analysis is a robust quantitative research technique designed to synthesize the statistical results of multiple empirical studies that investigate similar theoretical relationships. By aggregating effect sizes across studies, meta-analysis gives a thorough look at the strength, nature, and consistency of variable relationships, while also correcting for potential sampling error and publication bias. This method is particularly useful in fields with mixed results or fragmented theories, because it helps to identify overall patterns, spot inconsistencies, and find missing areas in the existing research.

To evaluate the potential influence of publication bias on meta-analytic findings, researchers often use the Fail-safe N approach, originally proposed by Rosenthal (1979), and Egger’s regression test, introduced by Egger et al. (1997). These methods address the file-drawer problem—where null results remain unpublished—and small-study effects, respectively, by assessing effect robustness and funnel plot asymmetry. The Fail-Safe N values are consistently large and statistically significant across all supported hypotheses, indicating that a substantial number of unpublished null studies would be required to render the observed effects non-significant, for example Fail-Safe N of H12 = 88.60; H14 = 42.85; H5 = 33.53. In addition, Egger’s regression intercepts are non-significant for all relationships (all p-values > 0.05), including those based on a large number of effect sizes, examples H12’s intercept value = −0.06, p = 0.99; H13’a intercept = 0.18, p = 0.95, suggesting no evidence of small-study effects or funnel plot asymmetry. Although substantial heterogeneity is present, the convergent results from these results indicate that publication bias is unlikely to materially affect the meta-analytic conclusions.

Within this research, meta-analysis functions as a robust tool for synthesizing the empirical findings about the factors that precede and result from fast fashion consumption, including PQ, behavioral control, SN, and their impacts on brand loyalty and WoM behavior. Following the protocols of Borenstein et al. (2010) and Wilson and Lipsey (2001), this meta-analytic investigation adheres to established guidelines to calculate pooled effect sizes, assess heterogeneity, and identify potential moderating influences. A random-effects model was employed throughout the study to account for differences between the research included, such as studied year, sample demographics, methodologies used, and cultural contexts ( Field & Gillett, 2010). The procedure ensures transparency, replicability, and theoretical advancement through the integration of dispersed findings.

### 3.2 Literature search

To compile a comprehensive dataset, the authors employed multiple search strategies across a variety of academic databases, including Scopus, Web of Science, ScienceDirect, JSTOR, SpringerLink, Emerald, and Google Scholar. The keywords used included combinations of terms such as ‘fast fashion,’ ‘purchase intention,’ ‘perceived quality,’ ‘brand loyalty,’ ‘behavioral control,’ ‘subjective norm,’ and ‘word of mouth.’ Additionally, the authors screened peer-reviewed journals focusing on marketing, consumer behavior, retail management, and fashion studies, such as the Journal of Retailing and Consumer Services, Journal of Fashion Marketing and Management, and Psychology & Marketing.

The search covered studies published between 2004 and 2024. Articles were excluded if they (a) were conceptual or purely theoretical, (b) lacked empirical data, (c) did not report the necessary statistical information (e.g., correlation coefficients, t-values, or standardized betas), or (d) focused on unrelated constructs. The research aimed to mitigate publication bias and the “file drawer problem” by including a variety of unpublished materials like conference papers, dissertations, and working papers ( Guzzo et al., 1987).

## 4. Results

The objective of this meta-analysis was to thoroughly examine sixteen theoretically valid hypotheses using frameworks such as the TPB, PS Theory, Self-Congruity Theory, PQ Theory, and the ECL Model (see Table 17).

**Table 17.** The meta-analysis results.

Effective size and 95% Confidence									Heterogeneity		
Variable			Interval								
Hyp	Ind.	Dep.	k	n	r	LCI	UCI	p-value	Q	χ *^2^*	*I ^2^*
H1	PS	AT	5	1431	0.334	0.234	0.427	0.000	16.883	9.488	76.308
H2	n/a	n/a	n/a	n/a	n/a	n/a	n/a	n/a	n/a	n/a	n/a
H3	n/a	n/a	n/a	n/a	n/a	n/a	n/a	n/a	n/a	n/a	n/a
H4	PS	IFFC	11	3600	0.181	0.044	0.311	0.010	174.041	18.31	94.254
H5	CFFB	AT	16	6328	0.420	0.314	0.515	0.000	95.747	25.00	95.747
H6	CFFB	SN	5	2934	0.544	0.408	0.656	0.000	91.841	9.49	95.645
H7	CFFB	PBC	4	2207	0.442	0.360	0.518	0.000	15.515	7.81	80.664
H8	PQ	AT	9	4537	0.509	0.345	0.643	0.000	353.394	15.51	97.736
H9	PQ	SN	3	1447	0.513	0.284	0.687	0.000	44.110	5.99	95.466
H10	PQ	PBC	5	4193	0.311	0.071	0.516	0.000	259.303	9.49	98.457
H11	PQ	IFFC	12	4270	0.351	0.221	0.468	0.000	258.706	19.68	95.362
H12	AT	IFFC	44	17760	0.605	0.491	0.698	0.000	5145.628	59.30	99.164
H13	SN	IFFC	34	13022	0.253	0.174	0.328	0.000	753.045	47.40	95.559
H14	PBC	IFFC	30	12788	0.385	0.264	0.494	0.000	1724.060	42.56	98.318
H15	IFFC	BL	6	1764	0.465	0.393	0.531	0.000	10.678	11.07	62.539
H16	IFFC	WOM	5	2009	0.560	0.240	0.770	0.000	304.288	9.49	98.685

The first set of hypotheses looked into the idea of PS, which comes from scarcity theory. H1 proposed a positive correlation between PS and consumer attitudes towards fast fashion. The meta-analytic estimate revealed a moderate effect size (r = 0.334, 95% CI [0.234, 0.427], p < 0.001), suggesting that limited availability likely augments consumers’ evaluative responses. Notable heterogeneity (Q = 16.883, df = 4, p = 0.002; I ^2^ = 76.31%) implies that this effect may be dependent on contextual variables, such as the manner in which scarcity cues are framed or perceived. These findings are congruent with both the S-O-R paradigm and the TPB, which posit that environmental stimuli can elicit urgency-driven attitudes.

H2 and H3, on the other hand, looked at how scarcity affects SN and PBC, but they couldn’t be evaluated because there weren’t enough qualifying primary research. Although the theoretical justification for these pathways remains plausible, particularly within digitally mediated contexts where social proof and peer validation amplify scarcity cues, their empirical validation is an endeavor for future research. This gap represents a significant blind spot in the extant literature.

H4 examined at how PS directly affects behavioral intention. The findings indicate a modest yet statistically significant effect (r = 0.181, 95% CI [0.044, 0.311], p 0.010), although substantial heterogeneity (Q = 174.041, df = 10, p < 0.001; I ^2^ = 94.25%) once more highlights the significance of moderating factors such as brand type, consumer characteristics, or the format of scarcity (e.g., time-limited versus quantity-limited). While scarcity seems to motivate purchase intention, its effects are notably heterogeneous.

H5 through H7 looked examined how the congruence between self-image and brand identity (CFFB) affects the main TPB constructs in the SC dimension. H5 revealed a robust correlation with consumer attitudes (r = 0.420, 95% CI [0.314, 0.515], p < 0.001), albeit accompanied by high heterogeneity (I ^2^ = 95.75%). Hypothesis 6 identified an even stronger relationship with SN (r = 0.544), underscoring the symbolic and social dimensions of brand identification. Similarly, H7 confirmed that SC significantly enhances PBC (r = 0.442, p < 0.001), indicating that alignment with brand identity strengthens consumers’ sense of agency. Across all three constructs, the variation among studies suggests that congruity is interpreted through culturally and psychologically situated identity frameworks.

H8 through H10 looked at the effect of the construct on the mediators of the TPB and focused on Perceived Quality Theory. H8 established a substantial and statistically significant positive correlation between PQ and attitudes (r = 0.509, 95% CI [0.345, 0.643], p < 0.001), although the heterogeneity was markedly pronounced (Q = 353.394, df = 8, p < 0.001; I ^2^ = 97.74%). H9 exhibited a comparable trend regarding SN (r = 0.513, 95% CI [0.284, 0.687], p < 0.001; Q = 44.110, df = 2, p < 0.001; I ^2^ = 95.47%), implying that product quality perceptions are not solely individualistic but may be shaped by social feedback and collective assessments. H10 found a statistically significant association with PBC (r = 0.311, 95% CI [0.071, 0.516], p < 0.001; Q = 259.303, df = 4, p < 0.001; I ^2^ = 98.46%). Overall, our data support the hypothesis that PQ has a major impact on cognitive evaluations that precede consumption intentions, albeit with considerable heterogeneity depending on contextual circumstances.

H11 expanded the area of PQ by exploring its direct impact on behavioral intentions. The study found a moderate, positive association (r = 0.351, 95% CI [0.221, 0.468], p < 0.001; Q = 258.706, df = 11, p < 0.001; I ^2^ = 95.36%), highlighting the importance of perceived product qualities in consumer decision-making. These findings are especially noteworthy in light of fast fashion’s aesthetic and trend-driven dynamics, in which quality is usually determined by surface indications such as style and digital display rather than actual endurance.

In the TPB framework, H12 found that attitude was the most important predictor of intention (r = 0.605, 95% CI [0.491, 0.698], p < 0.001), with significant heterogeneity (Q = 5145.628, df = 43, p < 0.001; I ^2^ = 99.16%). In H13, subjective norm had a substantial, albeit smaller, influence (r = 0.253, 95% CI [0.174, 0.328], p < 0.001; Q = 753.045, df = 33, p < 0.001; I ^2^ = 95.56%). H14 found a moderate impact of PBC on intention (r = 0.385, 95% CI [0.264, 0.494], p < 0.001), although heterogeneity remained high (Q = 1724.060, df = 29, p < 0.001; I ^2^ = 98.32%). These results validate the TPB’s structural integrity while highlighting varying degrees of influence, with evaluative attitudes having the greatest impact, followed by volitional control and social influences.

Finally, H15 and H16, which are based on the Expectation-Confirmation-Loyalty Model, studied the behavioral consequences of intention. H15 found a moderate and statistically significant relationship between intention and brand loyalty (r = 0.465, 95% CI [0.393, 0.531], p < 0.001), with a reduced level of heterogeneity (Q = 10.678, df = 5, p = 0.058; I ^2^ = 62.54%). This suggests a more consistent and generalizable relationship between intention and loyalty in different circumstances. H16 showed a stronger connection between intention and WoM behavior (r = 0.560, 95% CI [0.240, 0.770], p < 0.001), although heterogeneity was still significant (Q = 304.288, df = 4, p < 0.001; I ^2^ = 98.69%). These findings support the notion that behavioral intention serves not only as a cognitive predictor of action, but also as a precursor to relational and communicative consequences, such as brand advocacy and loyalty.

Fourteen out of sixteen hypotheses were empirically supported, with significant correlations (p < 0.001) and impact sizes ranging from small (r = 0.135) to large (r = 0.605). Attitude was identified as the most influential predictor of intention, whereas PQ and brand congruity had a significant impact across multiple dimensions of the TPB. The presence of heterogeneity in most relationships, as evidenced by significant Q-statistics and elevated I ^2^ values, suggests that future research should use moderator analyses (e.g., meta-regression, subgroup analysis) to better understand the discrepancies.

## 5. Discussion

Based on the Theory of TPB, Self-Congruity Theory, PQ Theory, and ECL Model, the present meta-analysis sheds light on a comprehensive view of the psychological and social determinants of consumer behavior in the fast fashion industry. Fourteen of the sixteen initially proposed hypotheses were supported by empirical evidence, revealing a wide range of effects and significant heterogeneity, thus emphasizing the robustness and contextual variability of the important predictors.

The results indicate that attitude is the strongest predictor of fast fashion consumption intention, corroborating the foundational premise of the original TPB that a consumer’s positive outlook is a major driver of their purchasing intentions. This study shows that attitude – a consumer’s overall positive or negative assessment of fast fashion – is an important internal factor that explains and guides how people behave as consumers in the fast fashion market where trends evolve quickly and buying choices are frequently based on emotion, impulse, or a product’s aesthetic charm.

Similarly, perceived quality is also an important factor influencing AT, SN, PBC, and IFFC, which affects multiple psychological and social dimensions simultaneously. These findings imply that consumers’ perceptions of product quality, whether symbolic (design or aesthetics) or practical (durability or material), are important in promoting social trust, behavioral control, and positive attitudes. This result confirms and extends Zeithaml’s theory (1988) in the context of perceived quality and value playing a key role in persuading consumers to shop frequently at low prices.

There was also a notable effect of CFFB on TPB’s key elements. The results show that when a brand aligns with a consumer’s self-image, they develop more favorable attitudes, a stronger sense of consumption control, and better social validation. These results confirm the extent to which product consumption is driven by identity, especially when the brand serves as a social display mechanism, and they support Self-Congruity Theory ( Sirgy, 1982).

Furthermore, PS had a moderate impact on AT and a small impact on IFFC, but the high heterogeneity of this factor suggests that it affects both contexts. Cultural differences may explain these contrasting results; while scarcity increases appeal in some markets, it may also be a factor that causes resistance or skepticism in others. Given these results, it is possible that cultural norms, consumer skepticism, or brand trust moderate the effect of scarcity on purchase behavior.

Although SN and PBC influence consumers’ intentions to participate in the fast fashion market to different degrees, the fact that both effects are supported further supports the TPB theory. Social pressure is also important yet, in the case of fast fashion, it is less influential than personal attitudes or perceptions of control, as evidenced by the smaller effect size associated with SN. On the other hand, PBC’s relative influence indicates that accessibility, affordability, and decision-making power are important considerations.

The final part of the research model, behavioral intention to effect, is also supported. Significant effects of IFFC are observed on both brand loyalty and WoM, supporting the post-consumption phase of the ECL model. Particularly for digital consumers, who share their choices with a significant portion of WoM influence, WOM becomes a tool of notable spillover potential in influencing purchase behavior.

In summary, the results support the important role of attitudes, perceived quality, and brand consistency in shaping fast fashion consumption, thereby reinforcing the TPB and related frameworks. However, the context-sensitive nature of the gap and the significant differences between studies highlight the need for market-specific approaches and additional empirical research.

### 5.1 Theoretical implications

By integrating these theoretical models, this study provides important findings theorized in key areas: existential formulation, personal identity, perceived quality, and the extension of the TPB to post-consumption behaviors.

First, this integration extends TPB theory by identifying external dimensions as antecedents to internal cognitive-affective states, particularly attitudes. Drawing on the S-O-R model ( Russell & Mehrabian, 1974), the analysis reveals that scarcity cues serve as theorized salient triggers that determine price evaluations and indirectly influence action intentions. This formulation is beyond the rational assumptions in the TPB that consumers are often experiential and emotionally arousing in high-speed consumer environments such as fast fashion.

Second, self-congruity helps to expand the psychological scope of the TPB model. The association between consumers’ self-concept and brand image significantly predicts not only attitudes but also SN and PBC, reinforcing the centrality of symbolic consumption in identity construction ( Sirgy, 1982; Gollwitzer et al., 1982). Combining this theory with social identity theory ( Tajfel & Turner, 1981) suggests that when consumers perceive a brand as reflecting their intrinsic values, normative pressure - and the perceived ease of performing those behaviors - increases. Thus, self-congruity suggests that personal identity is an important determinant of consumer intention.

Third, the findings challenge the traditional TPB construct by the cross-cutting influence of PQ ( Zeithaml, 1988) in the model. PQ significantly influences attitudes, SN, and PBC. This suggests that these factors are not limited to shaping outcome evaluations but also influence group identity or social status. PQ includes not only physical indicators such as durability and design but also ethical aspects such as sustainability, further complicating its evaluative role. Therefore, the results provide evidence in favor of a reconceptualization and multidimensionality of PQ, including cognitive, affective, and normative aspects.

Finally, the study extends the boundaries of the TPB by linking intentions to post-consumption outcomes—specifically, loyalty to cues and WoM. While TPB theory and its extensions typically rest on intentions or behavioral actions, loyalty and WoM in this study argue that intentions act as an antecedent to effective engagement and long-term social performance. These findings need to be viewed as a pivotal point in a chain of actions rather than an end point.

Overall, these contributions underscore the importance of integrating identity, emotion, environmental stimuli, and post-consumption motivation into action models. Future refinements of the TPB need to take into account the complexities of symbolism and context, especially in markets where consumption serves both objective and expressive purposes.

### 5.2 Practical implications

This research provides a series of practical insights that can help brands more quickly and effectively craft their next strategy. At the heart of these recommendations is a complex set of consumer perceptions—scarcity, identity congruence, perceived quality, and sense of behavioral control—that together shape how consumers interact with brands, make purchase decisions, and share their experiences with others.

First, perceived scarcity is a powerful marketing tool when used skillfully. Customers feel a sense of urgency and are motivated to make a purchase—a feeling similar to FOMO—when brands communicate scarcity by limiting the time of a promotion or the number or variety of products on shelves. However, this technique also has an impact on the brand if the customer does not feel the authenticity but feels psychologically manipulated, then the brand’s reputation will be affected. In markets where social factors have a strong impact, emphasizing the popularity of the product becomes a stimulus for purchase; conversely, in consumer markets where individuality or personal identity is promoted, being different or exclusive becomes an advantage to attract consumers.

Second, an important and valuable concept is the concept of self-congruity - a brand’s image is consistent with how consumers see themselves or want to be seen. This connection can have a profound effect on attitudes and behavior, especially in the world of fashion, where clothing often serves as an expression of self-expression. Brands that reflect consumers’ personal values and identities – through intimate stories, influencer partnerships, or customization opportunities – are more likely to build lasting emotional relationships. Younger, more marginalized consumers like Generation Z, in particular, respond strongly to campaigns that reflect their evolving sense of self and digital maturity. However, a one-size-fits-all message is not enough; cultural and generational differences need to be considered when defining identity-driven content.

Perceived quality also plays a key role in building consumer trust in all products and commodities, including fast fashion. This perception of quality goes beyond the fabric, fit, and stitching—it also includes how the product meets consumer expectations, such as how it makes the wearer feel, or even the social or environmental value associated with the brand. Quality signals can be both self-image-consistent and symbolic. Feedback from the community—in the form of reviews or endorsements from influencers—is important in enhancing perceptions of quality and trustworthiness, especially among highly socially connected consumers. It is a form of social validation that shapes purchase decisions in digitally-driven, community-driven marketplaces.

Ultimately, when consumers feel confident in their ability to make choices, navigate through options, and return items if necessary, they are more likely to make repeat purchases. This is achieved by giving consumers a greater sense of control over the buying process. Streamlining the customer experience – through user-friendly shopping interfaces, product personalization, and clear purchase, warranty, or return policies – can reduce the psychological barriers to purchase. For example, through digital platforms, using AI, businesses can help customers enhance their shopping experience with virtual fitting rooms, data-driven advice based on analysis of past customer shopping habits. This in turn, increases customers’ sense of control over their behavior in a powerful way.

Overall, the findings of this study demonstrate the value of integrating marketing approaches from making appeals to increase the perception of genuine scarcity, to building a brand based on identity, communicating a consistent quality message, and empowering consumers to make decisions. When tailored to each cultural context and customer segment, these strategies can help brands create unique consumer experiences, increase loyalty, and build effective brands. In a market with rapid, constant change and short consumption cycles, brands need to demonstrate flexibility and a continuous response strategy. Rapid adjustment to consumer behavior and cultural cues is key to maintaining competitive advantage and brand presence in the marketplace.

### 5.3 Limitations and future research directions

Despite its positive findings, the study has certain limitations. Grasping these limitations is key for providing future researchers with a roadmap for further investigation and model enhancement. An obvious limitation is shown by the significant heterogeneity among many hypotheses, which suggests that the effect sizes are inconsistent. This limitation suggests the presence of unaccounted-for moderators such as culture, psychology, or context, which affect the generalizability and theoretical precision of the findings. In the future, researchers could use meta-analytic regression or subgroup analysis to identify and quantify such moderators. Second, although recognizing potential moderators (e.g., cultural orientation, materialism, generational identity), the current study did not systematically examine their moderating effects. Addressing this gap would enable deeper insights and support context-specific strategic recommendations. Although the meta-analysis includes data from different national contexts, it lacks explicit modeling of cultural variables (e.g., collectivism, power distance). Future research should integrate established cultural frameworks to further elucidate how culture shapes consumer responses and moderates key relationships. Finally, the model ignores ethical and sustainability constructs, which have received increasing attention in fast fashion. Despite some implications for environmentally conscious behavior, sustainability has not been systematically and explicitly integrated into the research model. Future research should incorporate sustainability-related attitudes and values to enhance relevance in ethically conscious markets and reflect changing consumer expectations.

## Informed consent statement

This research is a meta-analysis and did not involve direct interaction or data collection from human participants. Informed consent was obtained in the primary studies included in this synthesis, as applicable to their research designs and data collection methods.

## Data Availability

All data underlying this systematic review and meta-analysis are openly available in Figshare at https://doi.org/10.6084/m9.figshare.30219034.v2 ( Hsu et al., 2025), under a CC0 license. The repository contains:
• Extracted study-level information, including sample sizes, effect sizes (correlations, regression coefficients), and study descriptors (e.g., country, method, demographics); • Pooled meta-analytic results with effect sizes, confidence intervals, and significance levels; • Heterogeneity statistics (Q, χ ^2^, I ^2^) for each hypothesis; • The completed PRISMA 2020 checklist; • The PRISMA 2020 flow diagram.
