# Supplementary material for: Psychological Drivers and Behavioral Outcomes of Fast Fashion Consumption: A Meta-Analytic
Source: F1000Res. 2026 Feb 10;14:1256. Originally published 2025 Nov 14. [Version 2] doi: 10.12688/f1000research.170388.2 (PMC12993291; doi:10.12688/f1000research.170388.2)
Supplement: Peer review history

# Reviewer report: Reviewer response for version 2

**DOI:** 10.5256/f1000research.194662.r457879

**Published:** 2026-03-16

**Authors:** Pablo Gutiérrez-Rodríguez

**Affiliations:** 1 Universidad de Léon, León, Spain

The manuscript addresses a timely and relevant topic and presents an ambitious theoretical integration combining the Theory of Planned Behavior with key brand-related constructs in the fast fashion context. The rationale and objectives are clearly articulated, and the use of a random-effects meta-analytic model is, in principle, appropriate given the diversity of cultural settings and empirical designs included in the analysis.

 However, in order for the manuscript to achieve full scientific robustness, several methodological and interpretative aspects should be strengthened. In particular, the methods section would benefit from greater transparency and detail regarding the search strategy, inclusion and exclusion criteria, study selection process, and the handling of potential dependencies among effect sizes. Although heterogeneity is acknowledged, a deeper exploration of its sources would enhance the credibility of the findings. Additionally, some conclusions adopt a tone that approaches causal interpretation, whereas the underlying evidence is primarily correlational; these implications should be framed more cautiously.

 Importantly, these observations do not undermine the viability of the manuscript but rather point to areas that are clearly addressable. With improved methodological transparency and a more carefully calibrated interpretation of the results, the manuscript has strong potential to become a rigorous and valuable contribution to the literature on consumer behavior in the fast fashion sector.

Are the rationale for, and objectives of, the Systematic Review clearly stated?

Yes

Is the statistical analysis and its interpretation appropriate?

Partly

If this is a Living Systematic Review, is the ‘living’ method appropriate and is the search schedule clearly defined and justified? (‘Living Systematic Review’ or a variation of this term should be included in the title.)

Yes

Are sufficient details of the methods and analysis provided to allow replication by others?

Partly

Are the conclusions drawn adequately supported by the results presented in the review?

Yes

Reviewer Expertise:

Marketing

I confirm that I have read this submission and believe that I have an appropriate level of expertise to confirm that it is of an acceptable scientific standard.

# Reviewer report: Reviewer response for version 2

**DOI:** 10.5256/f1000research.194662.r456981

**Published:** 2026-02-19

**Authors:** Tiara Nur Anisah

**Affiliations:** 1 Janabadra University, Yogyakarta, Indonesia

I have reviewed the revised manuscript (Version 2) and the authors' detailed responses. I appreciate the effort put into addressing the previous comments, particularly the inclusion of the Publication Bias Assessment and the availability of the PRISMA flow diagram in the repository. These additions have significantly improved the transparency of the study.

 Regarding the issue of high heterogeneity (I2), I accept the authors' explanation and the decision to acknowledge this in the 'Limitations and Future Research' section. While a subgroup analysis would have been ideal, listing it as a limitation is an acceptable compromise for the current scope of this meta-analysis.

 One minor technical note for the final version:

 In the newly added *Publication Bias* section, you cited a Fail-Safe N of 88.60 for H12. Strictly speaking, with k=44 studies, this value falls slightly below the classic Rosenthal threshold (5k+10, which would be roughly 230). However, since your *Egger’s regression test* results are non-significant (p > 0.05), the overall conclusion that there is no serious publication bias remains valid. You might want to slightly temper the phrasing "consistently large" to something more moderate in the final proof to be statistically precise, though this does not affect my final decision.

Are the rationale for, and objectives of, the Systematic Review clearly stated?

Yes

Is the statistical analysis and its interpretation appropriate?

Yes

If this is a Living Systematic Review, is the ‘living’ method appropriate and is the search schedule clearly defined and justified? (‘Living Systematic Review’ or a variation of this term should be included in the title.)

Not applicable

Are sufficient details of the methods and analysis provided to allow replication by others?

Partly

Are the conclusions drawn adequately supported by the results presented in the review?

Yes

Reviewer Expertise:

green & sustainability, marketing management, and digital marketing.

I confirm that I have read this submission and believe that I have an appropriate level of expertise to confirm that it is of an acceptable scientific standard.

# Reviewer report: Reviewer response for version 1

**DOI:** 10.5256/f1000research.187836.r433498

**Published:** 2025-12-01

**Authors:** Tiara Nur Anisah

**Affiliations:** 1 Janabadra University, Yogyakarta, Indonesia

**PEER REVIEW REPORT**

**Manuscript Title:** Psychological Drivers and Behavioral Outcomes of Fast Fashion Consumption: A Meta-Analytic

**Overall Evaluation:**

 This meta-analytic study is a highly relevant and timely contribution to the consumer behavior literature, specifically in the fast fashion context. The methodology, a random-effects meta-analysis, is appropriate for synthesizing a heterogeneous body of literature and has provided statistically robust effect sizes for the proposed conceptual model. The integration of the Theory of Planned Behavior (TPB) with brand-related antecedents (Self-Congruity, Perceived Quality, and Perceived Scarcity) is a significant theoretical extension. The manuscript is well-structured, and the results clearly support the majority of the hypotheses. However, two critical areas—high statistical heterogeneity and data limitations—require substantial methodological and discussion enhancements before acceptance.

**Detailed Review and Questions**

 1. Are the rationale for, and objectives of, the Systematic Review clearly stated?

**Yes**

 The rationale is clearly articulated by identifying significant gaps in the extant literature, including the siloed nature of single studies, the empirical fragmentation of TPB mediators, and the absence of a comprehensive synthesis. The three main objectives—to evaluate effects, examine mediating roles, and generate generalizable understanding—are also clearly presented.

 2. Are sufficient details of the methods and analysis provided to allow replication by others?

**Partly**

 The methodological foundation is sound, citing the random-effects model and adherence to Borenstein et al. (2010) and Wilson and Lipsey (2001) protocols. Details on the search strategy, including databases (Scopus, Web of Science, ScienceDirect, etc.), keyword groupings, and date range (2004–2024), are provided. 

**CRITICISM:** To ensure full replicability, the authors must provide the *specific search strings* used for each database and a formal reporting flow chart, such as a PRISMA diagram. Furthermore, details on the assessment or correction for publication bias (e.g., Egger's test, trim-and-fill procedure), which is a crucial component of robust meta-analysis, were mentioned as part of the protocol but not explicitly reported in the Results section.

 3. Is the statistical analysis and its interpretation appropriate?

**Yes**

 The statistical analysis is technically appropriate. But the authors must conduct and report a meta-regression or subgroup analysis (e.g., by culture, fast fashion brand type, or consumer demographic) to investigate the source of this heterogeneity. Without this step, the aggregated effect sizes ( *r* values) may mask significant underlying differences and limit the specificity of the theoretical and managerial conclusions. Additionally, the full model test is compromised by the inability to evaluate H2 and H3 due to a lack of data.

 4. Are the conclusions drawn adequately supported by the results presented in the review?

**Yes**

 The core conclusions are well-supported by the quantitative findings. For instance, the conclusion that Attitude is the most influential predictor of intention is directly supported by the largest effect size (r=0.605). Similarly, the robust influence of Self-Congruity (H5-H7, r ranging from 0.420 to 0.544) and the connection between intention and post-behavioral outcomes (H15 and H16, r=0.465 and r=0.560) are all clearly backed by the meta-analytic results.

 5. If this is a Living Systematic Review, is the ‘living’ method appropriate and is the search schedule clearly defined and justified?

**Not Applicable**

 This is a standard systematic review/meta-analysis, not a Living Systematic Review.

**Points that Must be Addressed to Make the Article Scientifically Sound (Critical Revisions):**

1. **Mandatory Meta-Regression/Subgroup Analysis:** The reporting of extremely high heterogeneity (I² = 95%) across most critical paths is a severe limitation on the generalizability of the pooled effect sizes. The authors must perform and report a meta-regression or subgroup analysis (e.g., utilizing 'Method/Country' or 'Studied Year' as a moderator from the source tables) to identify the specific variables causing this high variation. Without this, the overall effect sizes are minimally informative.
2. **Addressing Data Gaps and Full Model Test:** The authors should explicitly discuss the inability to test H2 (PS on SN) and H3 (PS on PBC). They must offer a theoretical justification for this 'blind spot' to guide future primary research (e.g., how the digital nature of fast fashion and 'fear of missing out' would theoretically mediate the social/control aspects).
3. **Enhancing Replicability:** The manuscript requires the inclusion of the full search strings used across the databases and a PRISMA flow chart detailing the study selection process (identification, screening, eligibility, inclusion) to meet rigorous reporting standards for systematic reviews.
4. **Reporting Publication Bias:** The authors must report the results of publication bias analysis (e.g., fail-safe N, Egger's test, or Trim-and-Fill) for the major relationships to confirm the robustness of the findings.

Are the rationale for, and objectives of, the Systematic Review clearly stated?

Yes

Is the statistical analysis and its interpretation appropriate?

Yes

If this is a Living Systematic Review, is the ‘living’ method appropriate and is the search schedule clearly defined and justified? (‘Living Systematic Review’ or a variation of this term should be included in the title.)

Not applicable

Are sufficient details of the methods and analysis provided to allow replication by others?

Partly

Are the conclusions drawn adequately supported by the results presented in the review?

Yes

Reviewer Expertise:

green & sustainability, marketing management, and digital marketing.

I confirm that I have read this submission and believe that I have an appropriate level of expertise to confirm that it is of an acceptable scientific standard, however I have significant reservations, as outlined above.

# Response

**Published:** 2025-12-31

**Authors:** KHEMRAJ SHARMA

**Affiliations:** Kalinga Institute of Industrial Technology, Bhubaneswar, Odisha, India

Reviewer Comments’ Response

 Points that Must be Addressed to Make the Article Scientifically Sound (Critical Revisions)

 1. Mandatory Meta-Regression/Subgroup Analysis: The reporting of extremely high heterogeneity (I² = 95%) across most critical paths is a severe limitation on the generalizability of the pooled effect sizes. The authors must perform and report a meta-regression or subgroup analysis (e.g., utilizing 'Method/Country' or 'Studied Year' as a moderator from the source tables) to identify the specific variables causing this high variation. Without this, the overall effect sizes are minimally informative.

**Response:**

 Thank you for this important comment.  We recognized that some I ^2^ is higher than 0.95 which indicate high heterogeneity in some hypotheses. Following McFadzean’s, et al.(1997) argument “..If this test shows homogeneous results then the differences between studies are assumed to be a consequence of sampling variation, and a fixed effects model is appropriate. If, however, the test shows that significant heterogeneity exists between study results then a random effects model is advocated...Although there is no statistical solution to this issue, heterogeneity between study results should not be seen as purely a problem for meta­analysis - it also provides an opportunity for examining why treatment effects differ in different circumstances…”We have used random effects analysis. Given that high heterogeneity may cause high variation. We have stated it **in 5.4 Limitation and Future Research Directions**:

 “Despite its positive findings, the study has certain limitations. Grasping these limitations is key for providing future researchers with a roadmap for further investigation and model enhancement. An obvious limitation is shown by the significant heterogeneity among many hypotheses, which suggests that the effect sizes are inconsistent. This limitation suggests the presence of unaccounted-for moderators such as culture, psychology, or context, which affect the generalizability and theoretical precision of the findings. In the future, researchers could use meta-analytic regression or subgroup analysis to identify and quantify such moderators. Second, although recognizing potential moderators (e.g., cultural orientation, materialism, generational identity), the current study did not systematically examine their moderating effects. Addressing this gap would enable deeper insights and support context-specific strategic recommendations. Although the meta-analysis includes data from different national contexts, it lacks explicit modeling of cultural variables (e.g., collectivism, power distance). Future research should integrate established cultural frameworks to further elucidate how culture shapes consumer responses and moderates key relationships”.

 2. Addressing Data Gaps and Full Model Test: The authors should explicitly discuss the inability to test H2 (PS on SN) and H3 (PS on PBC). They must offer a theoretical justification for this 'blind spot' to guide future primary research (e.g., how the digital nature of fast fashion and 'fear of missing out' would theoretically mediate the social/control aspects).

**Response**:

 We appreciate the reviewer’s observation regarding the inability to test **H2 (PS → SN)** and **H3 (PS → PBC)**. The exclusion of these hypotheses is due to insufficient empirical evidence: only one primary study was available for each relationship. Previous researches, such as Valentine et al. (2010) and Zaccagnini et al. (2023), stated that researchers need a minimum of 2 similar studies to perform a meta-analysis, because at least two sources of data are required to compute an aggregated effect size; a single study cannot provide a pooled summary statistic. Although we are unable to do meta analysis for H2 and H3, we have developed theses two hypotheses based on theoretical underpinnings. These two hypotheses, together with other hypotheses will be empirically tested again by survey data afterwards.

 3. Enhancing Replicability: The manuscript requires the inclusion of the full search strings used across the databases and a PRISMA flow chart detailing the study selection process (identification, screening, eligibility, inclusion) to meet rigorous reporting standards for systematic reviews.

**Response:**

 We thank the reviewer for emphasizing transparency and replicability. The **PRISMA flow diagram and checklist**, including detailed information on study identification, screening, eligibility, and inclusion, have been prepared and made publicly available as an independent supplementary report. The materials can be accessed via the following repository:

https://figshare.com/articles/dataset/Fast_Fashion_Consumption/30219034?file=58298377

 A reference to this repository has been added to the manuscript as an editor’s requirement to ensure full compliance with systematic review reporting standards. Based on editor’s comments, the Prisma flow diagram and checklist will be excluded from the manuscript but only shown as an independent supplementary report.

 4. Reporting Publication Bias: The authors must report the results of publication bias analysis (e.g., fail-safe N, Egger's test, or Trim-and-Fill) for the major relationships to confirm the robustness of the findings.

**Response:**

 Thank you for this valuable suggestion. In response, we have revised **Table 17** and added a formal **publication bias** **assessment** using **Fail-Safe N** (Rosenthal, 1979) and **Egger’s regression** **test** (Egger et al., 1997) to evaluate the robustness of the meta-analytic findings. Based on this table, the following paragraph is added to explain the result of publication bias. It was shown that publication bias is acceptable.

**Publication Bias Assessment**

 To evaluate the potential influence of publication bias on meta-analytic findings, researchers often use the Fail-safe N approach, originally proposed by Rosenthal (1979), and Egger’s regression test, introduced by Egger et al. (1997). These methods address the file-drawer problem—where null results remain unpublished—and small-study effects, respectively, by assessing effect robustness and funnel plot asymmetry. The Fail-Safe N values are consistently large and statistically significant across all supported hypotheses, indicating that a substantial number of unpublished null studies would be required to render the observed effects non-significant, for example Fail-Safe N of H12=88.60; H14=42.85; H5=33.53. In addition, Egger’s regression intercepts are non-significant for all relationships (all p-values > 0.05), including those based on a large number of effect sizes, examples H12’s intercept value= −0.06, p=0.99; H13’a intercept=0.18, p=0.95, suggesting no evidence of small-study effects or funnel plot asymmetry. Although substantial heterogeneity is present, the convergent results from these results indicate that publication bias is unlikely to materially affect the meta-analytic conclusions.
